# Editorial: Rising stars in pediatric cardiology 2023

**DOI:** 10.3389/fped.2024.1452884

**Published:** 2024-07-10

**Authors:** Liqun Sun, Laura Muiño-Mosquera

**Affiliations:** ^1^ Division of Cardiology, Department of Pediatrics, The Hospital for Sick Children, University of Toronto, Toronto, ON, Canada; ^2^ Translational Medicine Program, The Hospital for Sick Children, University of Toronto, Toronto, ON, Canada; ^3^ Division of Pediatric Cardiology, Department of Pediatrics, Ghent University Hospital, Ghent, Belgium; ^4^ Center for Medical Genetics, Ghent University Hospital, Ghent, Belgium

**Keywords:** pediatric cardiology, cardiovascular health, echocardiography, early stages, future innovations

**Editorial on the Research Topic** Rising stars in pediatric cardiology 2023

## Introduction

In this collection several researchers in the early stages of their careers present some of their latest works. This collection includes contributions across different aspects of pediatric cardiology.

## Fetal cardiovascular remodeling and post-natal cardiac function

Recent studies have demonstrated that different mechanisms leading to subclinical cardiac remodeling in fetal life, may predispose to clinical cardiac disease in childhood and adulthood after a second hit (1). Several of this mechanism have been postulated and include fetal growth restriction, preeclampsia, preterm birth, assist reproductive technology (ART), exposure to toxic drugs, twin-to-twin transfusion syndrome (TTTS) and maternal diabetes and obesity. Evidence suggests that ART induces alterations in cardiac and vascular morphology and physiology (2–5). In this collection, Li et al., conducted a comprehensive review of epigenetic mechanisms associated with an increased risk of cardiovascular disease (CVD) in offspring conceived via ART. Potential contributing factors include hormonal treatments, *in vitro* culture conditions, and epigenetic modifications. The findings underscore the significance of prenatal and postnatal monitoring of children conceived through ART, aiming to detect and address cardiovascular issues proactively associated with ART. The differences in DNA methylation might be due to aspects of ART procedures such as hormonal treatments, *in vitro* culture conditions, or other yet unknown factors. Reactive Oxygen Species (ROS) contribute to elevated oxidative stress in ART, potentially mitigated by antioxidants. Altered methylation patterns of genes, like AGTR1 and eNOS, are implicated in blood pressure abnormalities, with the ROS inhibitor melatonin preventing hypermethylation of eNOS (Li et al.).

## Congenital heart disease in the context of non-cardiovascular conditions

Congenital heart disease (CHD) encompasses a range of cardiac malformations present from birth, significantly impacting heart function. The prognostic outcomes in CHD are heterogeneous; certain malformations can be surgically corrected, resulting in near-normal life expectancy and quality of life. Individuals living with CHD frequently confront an elevated risk of subsequent cardiac complications, necessitating lifestyle adjustments and vigilant monitoring to prevent further cardiac deterioration. In this collection, Aly et al. studied the incidence of severe CHD in premature children and the impact of prematurity on outcome of children with severe CHD. The authors retrospectively examined a National multicenter database. They found a higher risk of severe CHD in premature infants from less affluent backgrounds. The study also examined the mortality rates in severe CHD cases vs. gestational age- matched controls and show increased major neonatal morbidity including higher rates of necrotizing enterocolitis, bronchopulmonary dysplasia, interventricular hemorrhage and periventricular leukomalacia adjusted for relevant factors such as birth weight and gender (Aly et al.). In another manuscript, Huang et al., conducts a study focusing on the importance of considering non-cardiac anomalies (NCAs) in children with CHD, as these can significantly impact the overall prognosis and management of the condition. Recognizing the varied probabilities and types of NCAs across different CHD subtypes is crucial for early detection, comprehensive evaluation, and tailored therapeutic approaches. The findings underscore the need for multidisciplinary teamwork involving cardiologists, geneticists, and other specialists to optimize care for these patients (Huang et al.). From the same group, Zhao et al., presented their comprehension of the complex interplay between primary ciliary dyskinesia (PCD), heterotaxy, and CHD, but it also highlights the necessity for preoperative screening of ciliary dysfunction in patients with heterotaxy and CHD. This proactive strategy can substantially improve postoperative respiratory care and ultimately enhance the overall health and well-being of these patients (Zhao et al.). Another example of the importance of considering NCAs in children with CHD is the study of Peng et al., which emphasizes importance of considering Tuberous Sclerosis Complex (TSC) in fetus and children with ardiac rhabdomyomas, regardless of size or location. Concurrent TSC leads to a generally less favorable prognosis due to epilepsy and neurological abnormalities, also granting a specialized and multidisciplinary approach (Peng et al.).

## Cardiovascular outcome in children with acquired cardiovascular diseases

Mu et al., devised a nomogram model specifically for patients diagnosed with Diffuse Large B-Cell Lymphoma (DLBCL). This model demonstrated admirable predictive efficacy and exceptional discriminative capability. Such a tool may substantially assist clinicians in formulating refined therapeutic strategies at the time of the initial diagnosis. The predictors identified for cardiovascular mortality (CVM) encompassed age at diagnosis, gender, ethnicity, tumor grade, Ann Arbor staging, receipt of radiotherapy, and specific chemotherapy regimens. Clinical variables ascertained at the diagnostic juncture can discern DLBCL patients who are at a heightened risk of CVM, thereby suggesting that preventative interventions should be contemplated for this subpopulation (Mu et al.).

In a multicenter prospective observational study, De Wolf et al., report on the late cardiac outcomes of 36 children who were recruited during the acute phase of multisystem inflammatory syndrome. In the late-term follow-up visit, the evaluation of late cardiac outcomes CMR does not show any myocardial scarring in children with a normal echocardiographic LVEF. Subclinical myocardial damage can persist in the late term, and further follow-up seems appropriate in these patients (De Wolf et al.).

## Illustrative case reports

This collection includes three interesting case reports illustrating different aspect of pediatric cardiology care. In the first case Luo et al., present a fetus with prenatal diagnosis of severe pulmonary stenosis with intact ventricular septum. In this case report, while the right ventricular volume did not reach significant values to support prenatal intervention, the authors observed a significant decrease in tricuspid valve gradient and annular z-score. Postnatally, a 1.5 ventricular circulation seemed the only feasible option. The authors concluded that besides right ventricular volume, which is the most accepted parameter for biventricular repair (PMID: 30238627), tricuspid valve regurgitation and z-score could be useful additional parameters.

In the second case report, Xu et al., present a child with excessive vagal tone manifesting as sinus pauzes of up to 7,4 s, multiple episodes of sinus bradycardia, and junctional escape rhythms. The authors apply for the first time in a child cardioneuroablation (CNA) therapy for this indication and demonstrate improved clinical outcomes, including symptom alleviation and resolution of rhythm disorders (Luo et al.). This innovative approach seemed therefor safe and effective.

Finally, in the last case report, Zhou et al., show a singular case of an infant diagnosed with Williams-Beuren syndrome (WS) who manifested an accelerated progression of arterial stenosis and exhibited left ventricular endocardial calcification. This case was associated with a novel heterozygous deletion not previously described in the literature. While arterial stenosis represents the most frequently encountered cardiovascular complication in patients with WS, the occurrence of endocardial calcification during infancy is exceptionally uncommon. To our knowledge, this is the first documented instance of endocardial calcification in an infant with WS, suggesting a unique phenotype that expands the known cardiovascular manifestations associated with this genetic condition (Zhou et al.).

## Conclusions

This collection gathers some interesting aspect of pediatric cardiology: (1) The study of cardiovascular remodeling during fetal life and its sequelae demands a multidisciplinary effort, encompassing developmental biology, genetics, epidemiology, and clinical cardiology. (2) A broad understanding of the molecular and environmental determinants of cardiovascular health and of disease associated with congenital and acquired heart diseases, will facilitate the development of innovative preventive, diagnostic, and therapeutic strategies. As research progresses, it is imperative that findings are translated into clinical practices that can mitigate the onset and progression of cardiovascular diseases, ultimately enhancing the quality of life and longevity for those affected.
